# *Plasmodium falciparum* Infection Induces Expression of a Mosquito Salivary Protein (Agaphelin) That Targets Neutrophil Function and Inhibits Thrombosis without Impairing Hemostasis

**DOI:** 10.1371/journal.ppat.1004338

**Published:** 2014-09-11

**Authors:** Michael Waisberg, Alvaro Molina-Cruz, Daniella M. Mizurini, Nidhi Gera, Beatriz C. Sousa, Dongying Ma, Ana C. Leal, Tainá Gomes, Michalis Kotsyfakis, José M. C. Ribeiro, Jan Lukszo, Karine Reiter, Stephen F. Porcella, Carlo J. Oliveira, Robson Q. Monteiro, Carolina Barillas-Mury, Susan K. Pierce, Ivo M. B. Francischetti

**Affiliations:** 1 Laboratory of Immunogenetics, National Institute of Allergy and Infectious Diseases, National Institutes of Health, Bethesda, Maryland, United States of America; 2 Department of Pathology, University of Virginia, Charlottesville, Virginia, United States of America; 3 Laboratory of Malaria and Vector Research, National Institute of Allergy and Infectious Diseases, National Institutes of Health, Bethesda, Maryland, United States of America; 4 Instituto de Bioquimica Médica, Federal University of Rio de Janeiro, Rio de Janeiro, Brazil; 5 Instituto de Ciências Biológicas e Naturais, Universidade Federal do Triângulo Mineiro, Uberaba, Brazil; 6 Institute of Parasitology, Academy of Sciences of the Czech Republic, České Budjovice, Czech Republic; 7 Research Technology Branch, National Institute of Allergy and Infectious Diseases, National Institutes of Health, Rockville, Maryland, United States of America; 8 Genomics Unit, Research Technology Section, Rocky Mountain Labs, National Institute of Allergy and Infectious Diseases, National Institutes of Health, Hamilton, Montana, United States of America; Institut Pasteur, France

## Abstract

**Background:**

Invasion of mosquito salivary glands (SGs) by *Plasmodium falciparum* sporozoites is an essential step in the malaria life cycle. How infection modulates gene expression, and affects hematophagy remains unclear.

**Principal Findings:**

Using Affimetrix chip microarray, we found that at least 43 genes are differentially expressed in the glands of *Plasmodium falciparum*-infected *Anopheles gambiae* mosquitoes. Among the upregulated genes, one codes for Agaphelin, a 58-amino acid protein containing a single Kazal domain with a Leu in the P1 position. Agaphelin displays high homology to orthologs present in *Aedes* sp and *Culex* sp salivary glands, indicating an evolutionarily expanded family. Kinetics and surface plasmon resonance experiments determined that chemically synthesized Agaphelin behaves as a slow and tight inhibitor of neutrophil elastase (*K_D_*∼10 nM), but does not affect other enzymes, nor promotes vasodilation, or exhibit antimicrobial activity. TAXIscan chamber assay revealed that Agaphelin inhibits neutrophil chemotaxis toward *f*MLP, affecting several parameter associated with cell migration. In addition, Agaphelin reduces paw edema formation and accumulation of tissue myeloperoxidase triggered by injection of carrageenan in mice. Agaphelin also blocks elastase/cathepsin-mediated platelet aggregation, abrogates elastase-mediated cleavage of tissue factor pathway inhibitor, and attenuates neutrophil-induced coagulation. Notably, Agaphelin inhibits neutrophil extracellular traps (NETs) formation and prevents FeCl_3_-induced arterial thrombosis, without impairing hemostasis.

**Conclusions:**

Blockade of neutrophil elastase emerges as a novel antihemostatic mechanism in hematophagy; it also supports the notion that neutrophils and the innate immune response are targets for antithrombotic therapy. In addition, Agaphelin is the first antihemostatic whose expression is induced by *Plasmodium* sp infection. These results suggest that an important interplay takes place in parasite-vector-host interactions.

## Author Summary

Malaria is transmitted by *Plasmodium falciparum*-infected *Anopheles gambiae* mosquitoes. Salivary gland contributes to the development of the parasite by creating a favorable environment for the infection and facilitating blood feeding and reproduction of the vector. However, the molecular mechanism by which the vector salivary gland modulates parasite/host interactions is not understood. We discovered that infection of the mosquito salivary gland upregulates several genes; among them, one codes for a protease inhibitor named Agaphelin. Notably, Agaphelin was found to exhibit multiple antihemostatic functions by targeting elastase. As a result, it inhibits platelet function which is required for blood to clot, and it prevents cleavage of TFPI, an anticoagulant that has recently been found to play a crucial role in thrombus formation *in vivo*. Agaphelin also attenuates neutrophils chemotaxis and the release of Neutrophil Extracellular Traps. These results provide evidence that neutrophils serve as a link between coagulation and the innate immune response. Agaphelin also exhibits anti-inflammatory and antithrombotic effects *in vivo*. Furthermore, Agaphelin did not promote bleeding, suggesting that targeting neutrophil exhibits potential therapeutic value. Altogether, these results highlight that the interplay between parasite, vector and host is a dynamic process that contributes and sustains the interface among *Plasmodium*, *Anopheles* and humans.

## Introduction

Hematophagous animals are strictly dependent on blood feeding for survival and reproduction. During feeding, the mouthparts of mosquitoes canulate or lacerate arterioles and venules, or penetrate hemorrhagic pools [1]–[3]. These events cause vascular injury, and the host response is accompanied by vasoconstriction, exposure of tissue factor (TF), endothelial cell injury, and activation of platelets, monocytes, and neutrophils [4]–[8]. Among these, neutrophils are particularly important in the early phase of inflammatory response and defense against infection. Accordingly, neutrophils phagocytose and kill intracellularly invading microorganisms in the phagosome by a mechanism involving reactive oxygen species, proteases, and antimicrobial peptides [4]–[8]. Evidences have also been provided that neutrophils can kill pathogens in an extracellular manner that does not require phagocytic uptake. This mechanism consists of web-like structures of DNA and proteins—known as neutrophil extracellular traps (NETs)—via a process called NETosis [4]–[8]. NETs released by activated neutrophils bind and kill pathogens. NETs are also important in inflammation and thrombus formation, as they operate as scaffolds to activate the extrinsic pathway via TF, the contact pathway via FXIIa, and platelet aggregation via histones [4]–[8].

Neutrophils also release granule contents containing enzymes such as elastase, proteinase-3, and cathepsin G. Elastase, a particularly inflammatory enzyme, interferes with thrombomodulin function [9], and cleaves TF pathway inhibitor (TFPI) [10], the physiological inhibitor of the extrinsic pathway [11]. More recently, it has been revealed that TFPI degradation by elastase plays a critical role in thrombosis *in vivo* [12], [13]. Elastase also induces proteinase-activated receptor activation, contributes to cytokine production and processing, inactivates metalloprotease inhibitors, degrades the extracellular matrix, promotes endothelial cell apoptosis and detachment, and affects leukocyte chemotaxis [14]–[16]. These events contribute to increased inflammatory and thrombotic tonus such as observed in ischemia reperfusion [17], acute myocardium infarction [6], venous thrombosis [18], [19], stroke [20] and cancer [21]. To regulate neutrophil function in general—and elastase activity in particular—a number of physiologic inhibitors of the enzyme have been described including serpins α-1 proteinase inhibitor and monocyte neutrophil elastase inhibitor (serpin B). In addition, elastase is inhibited by the chelonianin family of canonical inhibitors such as secretory leukocyte proteinase inhibitor (SLPI) and Elafin [22]. α2-Macroglobulin also inhibits elastase, among other enzymes. There is increasing evidence that these inhibitors, besides regulating inflammation by inhibiting proteolytic activity of proteases, also directly affect leukocyte chemotaxis and pro-inflammatory mediator release and may contribute to defense against invading pathogens. Therefore, elastase has been the target for several inhibitors (sivelestat, elafin, AZD9668) that have been tested under specific pathologic conditions associated with neutrophil dysfunction with mixed results according to different clinical trials [22].

In addition to physiologic inhibitors, bloodsucking arthropods exhibit an extensive repertoire of salivary molecules belonging to different family of proteins which target neutrophils, including protease inhibitors, chemokine-binding proteins and disintegrins which potentially affect inflammation [23]–[27]. However, direct blockade of neutrophil function by salivary proteins has not been shown to interfere with thrombosis *in vivo*, which is usually mediated by anticoagulants, platelet aggregation inhibitors, and vasodilators [2]. Here we demonstrate that the salivary gland (SG) of the malaria vector *Anopheles gambiae* presents several genes that are up- or down-regulated upon infection with *Plasmodium falciparum*, the main causative agent for severe malaria [28], [29]. Among the upregulated genes, we identified a putative antihemostatic peptide belonging to the family of Kazal type inhibitors. Agaphelin was found to inhibit elastase and proteinase-3, to modulate various neutrophil functions, and to prevent arterial thrombosis without impairing hemostasis. Agaphelin biological function emerges as a novel antihemostatic mechanism in hematophagy.

## Results

### Effects of *P. falciparum* on *An. gambiae* SG gene expression

We compared the gene expression of infected and uninfected SG tissues from *An. gambiae* mosquitoes. At least 43 genes were differentially expressed between infected and uninfected SGs; 5 (11.6%) were downregulated, while the remaining 38 (88.4%) were upregulated (Table 1). Among those upregulated were genes involved in chitin metabolism (AGAP002457), metal metabolisms (metallothioneins; AGAP001889 and AGAP001890), hemostasis (AGAP007907), antimicrobial humoral response (AGAP000999), regulation of saliva secretion and chemotaxis (AGAP002865), stress response (AGAP011970), lipid biosynthesis, transport (AGAP010973, AGAP002379, AGAP001185, AGAP002415), and signal transduction (AGAP001889), among others. The downregulated genes are related to housekeeping proteins and metabolism. To validate the microarray results, we performed nanostring RNA quantification for 19 *An. gambiae* genes. Overall, the microarray array and nanostring data presented a good degree of correlation (Pearson correlation coefficient of 0.95, with *P*<0.01).

**Table 1:** List of genes considered to be differentially expressed in salivary glands of *A. gambiae*, upon infection with *Plasmodium falciparum*.

*Ensembl ID*	*Best Hit to KOG*	*Best Match to Aedes Proteome*	*Biomart Description*	*SG-I/SG-C signal ratio* *
AGAP010911	Carboxylesterase and related proteins	carboxylesterase		113.89
AGAP004851	Uncharacterized conserved protein, contains laminin, cadherin and EGF domains	conserved hypothetical protein		58.53
AGAP000647		hypothetical protein		49.34
AGAP013359		conserved hypothetical protein		42.6
AGAP010360	Beta-spectrin	conserved hypothetical protein		42.52
AGAP010930	Protein tyrosine phosphatase	conserved hypothetical protein		33.82
AGAP007612		conserved hypothetical protein		33.09
AGAP006195		conserved hypothetical protein		32.83
AGAP011477	Trypsin	trypsin		29.71
AGAP006793	Semaphorins	conserved hypothetical protein		27.36
AGAP006416	Trypsin	serine-type endopeptidase,	Serine protease SP24D precursor (EC 3.4.21.-)	25.96
AGAP002865	Cytochrome P450 CYP3/CYP5/CYP6/CYP9 subfamilies	cytochrome P450	Cytochrome P450 CYP6P3	17.46
AGAP010458		conserved hypothetical protein		17.11
AGAP005501	Predicted dehydrogenase	oxidoreductase		16.26
AGAP006414	Chitinase	brain chitinase and chia	Chitinase	15.96
AGAP008218	Cytochrome P450 CYP3/CYP5/CYP6/CYP9 subfamilies	cytochrome P450	cytochrome P450	15.89
AGAP007907	Kazal type inhibitor (Agaphelin)	conserved hypothetical protein		15.32
AGAP010973	K+-dependent Ca2+/Na+ exchanger NCKX1 and related proteins	potassium-dependent sodium-calcium exchanger, putative		14.89
AGAP005524	WD40 repeat protein	conserved hypothetical protein		14.28
AGAP006194		conserved hypothetical protein		12.01
AGAP011970	Soluble epoxide hydrolase	epoxide hydrolase		11.51
AGAP012166	Phosphatidylinositol transfer protein SEC14 and related proteins	conserved hypothetical protein		11.18
AGAP009906		conserved hypothetical protein		9.09
AGAP012425	Membrane glycoprotein LIG-1	leucine-rich transmembrane protein		7.79
AGAP008923		conserved hypothetical protein		6.62
AGAP007974	C2H2-type Zn-finger protein	conserved hypothetical protein	Protein drumstick	6.53
AGAP009052		hypothetical protein		6.52
AGAP001890		conserved hypothetical protein	Metallothionein 2 (Fragment)	5.48
AGAP007386	G protein-coupled seven transmembrane receptor	lysozyme P, putative	Lysozyme c-7	5.09
AGAP008113		conserved hypothetical protein		4.73
AGAP000367	Predicted glyoxalase	lactoylglutathione lyase		4.5
AGAP009745	Predicted transporter (major facilitator superfamily)	sugar transporter		4.12
AGAP009745	Predicted transporter (major facilitator superfamily)	sugar transporter		3.99
AGAP001889		conserved hypothetical protein	Metallothionein 1 (Fragment)	3.91
AGAP006729	Uncharacterized conserved protein	conserved hypothetical protein		3.19
AGAP011603	Long-chain acyl-CoA synthetases (AMP-forming)	long-chain-fatty-acid coa ligase		2.63
AGAP002415	Predicted Rho GTPase-activating protein	conserved hypothetical protein		2.59
AGAP002457	Chitinase	brain chitinase and chia		2.57
AGAP006997	Alanyl-tRNA synthetase	alanyl-tRNA synthetase		0.6
AGAP002144	U4/U6 small nuclear ribonucleoprotein Prp4 (contains WD40 repeats)	wd-repeat protein		0.48
AGAP007851		conserved hypothetical protein		0.41
AGAP008096	Acid sphingomyelinase and PHM5 phosphate metabolism protein	sphingomyelin phosphodiesterase		0.33

*Genes considered to be differentially expressed in infected salivary glands (SG-Is) when compared with normal uninfected SGs (SG-C). Annotation of the probes considered to be differentially expressed was improved by searching Ensembl with Biomart and AnoxCel. Descriptions from KOG, Biomart, and the best match in the *A. aegypti* proteome are listed. SG-I:SG-C signal ratio represents the signal ratio of the infected to uninfected SG (n = 3 pools of mosquitos each, significance threshold is *p*<0.05 and fold change >2).

### Characterization of Agaphelin

**Figure 1:**
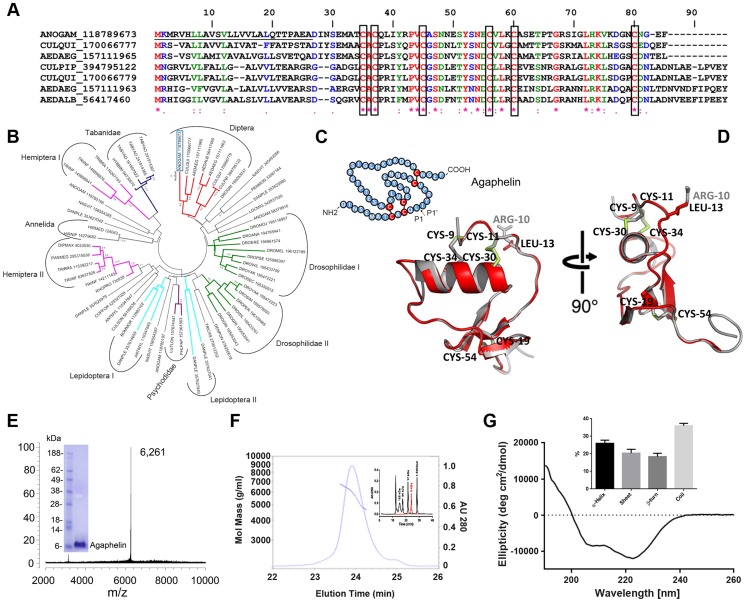
Characterization of Agaphelin. A) Clustal alignment of Agaphelin (gi| 118789673) with other proteins of the Kazal superfamily from Diptera. The boxes indicate the six conserved cysteines. Symbols below the alignment indicate: (*) identical sites; (:) conserved sites; (.) less-conserved sites. The underlined represent the signal peptide. B) Phylogram of Agaphelin (blue box) and other organisms obtained by the neighbor-joining algorithm using pairwise deletion and Poisson model. Sequences from the nonreduntant protein database of the National Center for Biotechnology Information (NCBI) are represented by the first three letters of their genus name, followed by the first three letters of the species name, followed by gi| accession number. Numbers in the phylogram nodes indicate percent bootstrap support for the phylogeny after 10,000 iterations. The bar indicates 10% amino acid divergence in the sequences. C) Secondary structure of Agaphelin [31], [34]. D) Structural model of Agaphelin (red) superimposed to Infestin 4 (gray). The six cysteines and the residue at position P1 from Agaphelin are labeled in black while the P1 residue of Infestin 4 is labeled in gray. Disulfide bonds are marked in yellow. E) Mass spectrometry analysis for Agaphelin. *Inset*: SDS/PAGE of Agaphelin, under reducing conditions. F) Analysis by SEC-MALS-HPLC provided the molar mass distribution of the main peak compared with absorbance at 280 nm. The continuous and interrupted lines represent absorbance 280 nm and MALS results, respectively. *Inset*: molecular weight markers (black lines) were loaded in the same column, and elution time was compared with Agaphelin (red line). G) Circular dichroism spectrum of Agaphelin showing a negative peak maxima at 222.6 nm and 208.0 nm and a positive peak maximum at 190 nm, indicating significant α-helix content. The ellipticity (degrees cm^2^/dmol) was plotted as a function of wavelength (nm) composition of Agaphelin. Data show the percentage of each type of secondary structure as determined by DichroWeb server (inset).

Among the genes that were upregulated in infected mosquito SG, we found one Kazal-type protease inhibitor, Agaphelin 15.32 time more expressed (gi118789673 GeneBank; AGAP007907, VectorBase database). Other anti-hemostatic proteins, such as the D7 family and apyrase did not reach the threshold indicative of up- or down-regulation. Agaphelin is also expressed in the midgut [30], but we did not find differential expression upon infection (microarray results and methods not shown). The Agaphelin gene is 255 bp long and encodes a 54 amino acid long mature protein containing a single Kazal inhibitory domain [31], with 6 cysteines and an estimated mol. wt. of 6.27 kDa and p*I* of 5.09. The protein contains a signal peptide predicted to be cleaved between residues AEA–DI, suggesting that it is secreted in the saliva. Figure 1A shows the alignment of Agaphelin with other Diptera family members.

The phylogenetic tree (Fig. 1B) indicates that Agaphelin and Kazal-type proteinase inhibitors belong to an expanded family of proteins from several Arthropoda organisms. Agaphelin has 41% identity with anticoagulant Infestin 4, an FXIIa inhibitor from *T. infestans* midguts (PDBID: 2ERW). It also shows a high level of identity with various other proteins with known crystal structures, including Anemonia elastase inhibitor (AEI) (35% identity, PDBID: 2LEO), 38% to Rhodniin, a specific thrombin inhibitor found in midguts of *R. prolixus* (PDBID 1TBQ), and to a thrombin inhibitor from *A. aegypti*. The Lepidoptera I clade clusters proteins are possibly related to defense against microorganisms and cocoon protection against predators, while a Kazal-type inhibitor from the black tiger shrimp *Penaeus monodon* showed inhibitory activity against subtilisin. The clade containing vasotab—a vasodilator from SGs of horse fly *H. bimaculata* [32]—and other sequences from Tabanidae has strong bootstrap support and clusters separately from Agaphelin, indicating different functions of proteins within the same family.

The predicted secondary structure of Agaphelin is presented in Fig. 1C. Within the Kazal domain, there are well conserved cysteine residues capable of forming three intradomain disulfide cysteine residues that can form disulfide bridges between cysteines 1–5, 2–4, and 3–6, resulting in a characteristic 3D structure [33]. Molecular homology modeling shows a high level of structural conservation of Agaphelin with other members of the Kazal family of inhibitors (Fig. 1D). It presents the characteristic structure of Kazal domains with a central α-helix inserted between two β-strands with a third one located toward the C-terminus. The reactive site P1 (located at position C_2_-X-P1) and the predicted conformation of the reactive site loop are structurally similar to those of other Kazal inhibitors, maintaining the canonical nature of the family. Agaphelin reactive site P1 contains a leucine (position 13) predicted to have activity against elastase, subtisilin, and chymotrypsin [34].

### Synthesis of Agaphelin

Agaphelin was chemically synthesized and refolded yielding a peptide that was >95% pure. Mass-spectrometry analysis indicates a mass of 6,261 Da (Figure 1E), which is in agreement with a predicted mass of 6,267 Da for refolded Agaphelin; it migrates as a ∼6 kDa protein in SDS/PAGE (inset). In addition, Agaphelin elutes as ∼6 kDa protein in gel-filtration chromatography and the light-scattering plot determined that it exhibits a hydrodynamic radius of 1.4 nm, suggesting a globular conformation (Figure 1F). By SEC, 87% of the protein elutes as a monomer, 4% as aggregates, and 8% as break-down products. Consistent with the structure of folded Kazal inhibitors and its predicted secondary structure, the CDS of Agaphelin showed negative peak maxima at 222.6 nm and 208.0 nm and a positive peak maximum at 190 nm, indicating significant α-helix content (Fig. 1G). Analysis of the experimental circular dichroism data using the DichroWeb server gave a composition of 26%±5% α-helix, 20%±7% β-sheet, 18%±6% β-turn, and 36%±4% random coil. The composition of the predicted homology models using the DSSP program gave a predicted composition of 17.5%±1.8% α-helix, 16.3±1.44% β-sheet, 11.3±2.98 β-turn, and 54.9±2.77% not assigned to any category by DSSP (Figure 1G, inset).

### Agaphelin is an inhibitor of elastase

**Figure 2:**
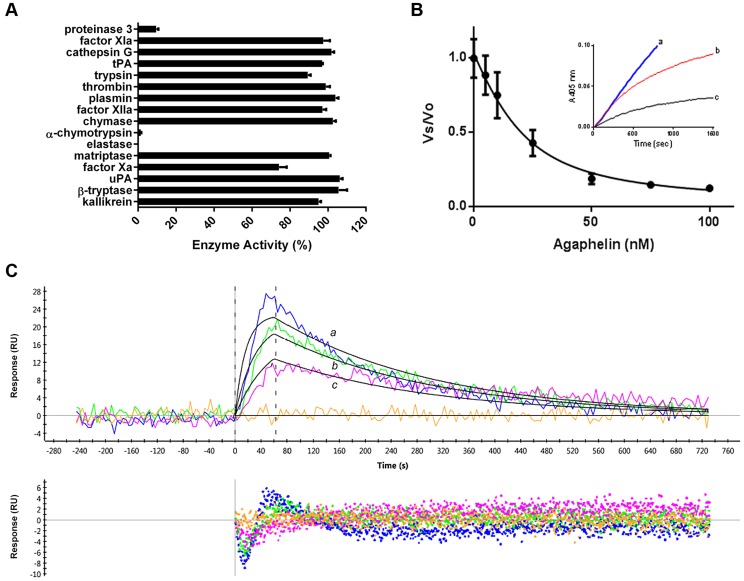
Agaphelin inhibits elastase. A) Agaphelin (1 µM) was tested against 16 different serine proteases in triplicates. Enzyme concentrations are provided in Material and Methods, (*, *t*-test; *p*≤0.05). B) Agaphelin inhibits elastase. Tight-type inhibitor: Elastase (18 nM) was incubated with Agaphelin (0–100 nM) for 30 min at RT followed by addition of chromogenic substrate MeOSuc-AAPV-pNA (600 µM). Residual activity was plotted as *Vo* (final velocity)/*Vs* (initial velocity). *Inset*: slow-type inhibitor: elastase (18 nM) was added to a mixture containing chromogenic substrate (600 µM) and Agaphelin (*a*, 0 nM; *b*, 150 nM, and *c*, 500 nM). C) Surface plasmon resonance experiments. Upper panel: Agaphelin was immobilized in a GLC chip and elastase was injected for 60 sec at 100 nM (*a*, blue), 50 nM (*b*, green), and 25 nM (*c*, pink). Dissociation of the Agaphelin-elastase complex was monitored for 600 sec. Representative sensograms (upper panel) are shown in black lines, and global fitting of the data points using the Langmuir equation is depicted. Lower panel: residual response.

Kazal-domain containing peptides are known to inactivate proteases [34]. Screening assays determined that Agaphelin inhibited elastase, proteinase-3, and chymotrypsin catalytic activity estimated with fluorogenic substrate (Fig. 2A). At large molar excess, it showed no or residual activity against other proteases involved with coagulation or inflammation (*i.e.*, cathepsin G, trypsin, chymase, matryptase, β-tryptase, kallikrein, urokinase-type plasminogen activator, FXa, FXIa, FXIIa, plasmin, thrombin, and tissue-type plasminogen activator). As a control, another synthetic salivary kazal-type inhibitor from *Triatoma infestans* (gi149898841) which does not exhibit Leu in the P1 position, did not inhibit elastase or any other enzyme (data not shown) Agaphelin (1.5 µM) did not prolong PT or aPTT, while the heparin control prolonged the aPTT (10 µg/ml) and the PT (100 µg/ml) (not shown). Figure 2B shows that incubation of elastase with Agaphelin is accompanied by blockade of the enzyme catalytic activity, with an *IC_50_* 4.46±0.03 nM. BotDB server calculations using Cheng and Prusoff equation determined a *Ki(app)* 9.22±0.8 nM, assuming a competitive type of inhibition [35]. In addition, Agaphelin behaves as a slow-type inhibitor, since addition of enzyme to a mixture containing substrate and inhibitor is accompanied by progress curves with a downward concavity. SPR experiments were performed where Agaphelin was immobilized in sensor chips followed by injection of elastase as analyte. Analysis of the sensorgrams using Langmuir equation determined *K_D_* 17.4±3 nM (Figure 2C).

Kazal-type inhibitors may also promote vasodilation or exhibit antimicrobial activity [34]. Agaphelin (up to 512 µg/ml, 81 µM) was devoid of antimicrobial activity (calculated by the MCI) toward *Staphylococcus epidermidis* (ATCC12228), *Enterococcus fecalis* (ATCC29212), *Escherichia coli* (ATCC 25922), *Klebsiella pneumonia* (ATCC 10031), *Proteus mirabilis* (ATCC 29245), *Serratia marcascens* (ATCC13880*), Acinetobacter baumannii* (MDR1674627), *Pseudomonas aeruginosa* (ATCC27835), and *Staphylococcus aureus* (MRSA33591). Controls with ciprofloxacin indicated a MIC of <1 µg/ml (not shown). Agaphelin (512 µg/ml) was also tested for MBC and did not show activity for *E. coli* (ATCC29212), *S. aureus* MRSA (33591), or *E. fecalis* (ATCC29212) (not shown). In addition, Agaphelin (1 µM) did not promote vasodilation of the rat aorta, while nitrophorin (1 µM), a nitric-oxide releasing molecule, produced a robust response (not shown).

### Agaphelin inhibits neutrophil chemotaxis

**Figure 3:**
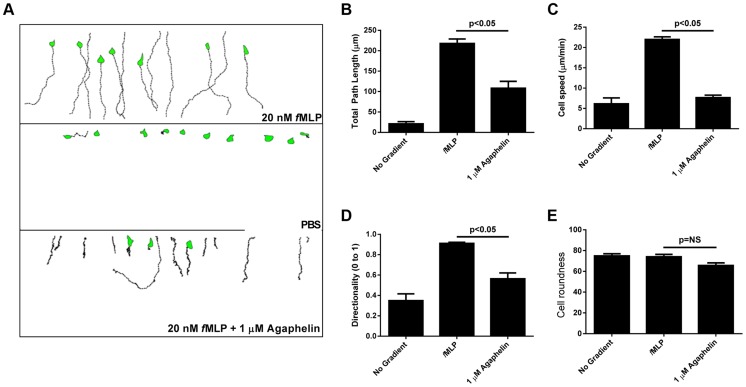
Agaphelin inhibits neutrophil chemotaxis *in vitro*. A) Chemotaxis of HL60 cells in EZ-TAXIScan chambers. Upper panel: HL60 cells incubated with PBS and then exposed to fMLP (20 nM) concentration gradient. Middle panel: HL60 cells incubated with buffer only and not exposed to *f*MLP. Lower panel: HL60 cells incubated with Agaphelin (1 µM) for 1 h and exposed to *f*MLP (20 nM). B–E) Quantification. Cell migration was analyzed based on the results presented in (A) and plotted for the following parameters: B) Total path length; C) Cell speed; D) Directionality, and E) Cell roundness. Data are expressed as the mean ± S.E (*, p≤0.05; *t*-test).

Inhibition of serine proteases may reduce neutrophil infiltration and neutrophil-mediated damage in various *in vivo* models of acute and chronic inflammation [15]. Agaphelin was tested on neutrophil chemotaxis toward *f*MLP using an EZ-TAXIScan chamber [36]. Figure 3A shows that Agaphelin is a potent inhibitor of neutrophil chemotaxis triggered by *f*MLP *in vitro*. Quantification of the results demonstrated that Agaphelin reduces several markers of chemotaxis including the total path length travelled by the cells (Fig. 3B), net path length (not shown), cell speed (Fig. 3C), and directionality (Fig. 3D) without affecting cell roundness (Fig. 3E).

### Agaphelin inhibits platelet aggregation, cleavage of TFPI and neutrophil-induced coagulation

**Figure 4:**
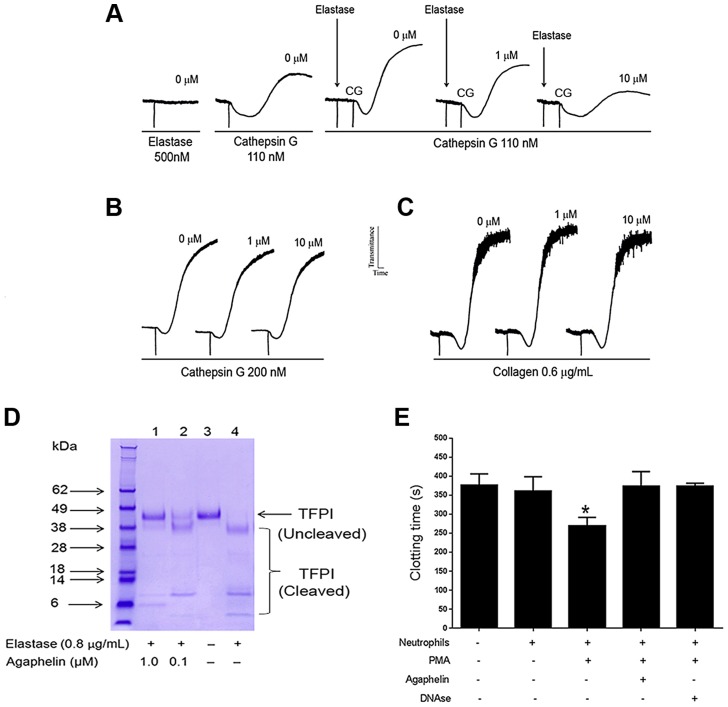
Agaphelin inhibits elastase-mediated platelet aggregation, TFPI-cleavage by elastase, and neutrophil-induced coagulation. A) Platelet aggregation. Washed human platelets were stimulated with elastase only (500 nM, left panel), or cathepsin G only (110 nM, middle tracing), or elastase followed by cathepsin G (right tracing). In some experiments, Agaphelin (1 or 10 µM) was added to platelets followed by addition of elastase for 1 min, and cathepsin G. B) Agaphelin does not inhibit cathepsin G (200 nM) or C) collagen (0.6 µg/ml)-induced platelet aggregation. Aggregation response was monitored by turbidimetry using a Lumi-Aggregometer. D) TFPI cleavage. Agaphelin (0.1 µM and 1 µM) was incubated with 1 µg of TFPI in the presence of PBS or human neutrophil elastase (0.8 µg/ml). After 2 h, reactions were stopped by addition of LDS loading buffer (under reducing conditions, 10 mM DTT), boiled for 5 min, and loaded in 4–12% Nu-PAGE gel. Gels were Coomassie Blue-stained. E) Neutrophil-induced coagulation. Neutrophils (5×10^5^ cells/well) were incubated with Agaphelin (1 µM), PBS (control) or DNAse (Dornase alfa, 4 µg/ml) for 1 hour, followed by addition of PMA (50 nM) for 3 hrs. Fifty µl of this suspension was added to 50 µl of plasma, and reactions were started by addition of CaCl_2_ (12.5 mM, final concentration)(*, p<0.05).

Neutrophils contribute to thrombosis through platelet aggregation, cleavage of TFPI, and release of NETs [4]. Elastase also plays a role in inflammation because it primes platelet aggregation induced by cathepsin [37]. Figure 4A shows that addition of elastase to platelets does not promote shape change or aggregation (left panel). On the other hand, low doses of cathepsin G activate platelets (middle panel). When elastase is added to platelets, followed by addition of cathepsin G, a robust aggregation is attained; however, when elastase was added to platelets previously incubated with Agaphelin, potentiation by cathepsin G no longer takes place (right panel). Control experiments demonstrated that Agaphelin did not affect cathepsin G-(Figure 4B) or collagen-induced platelet aggregation (Figure 4C). These results suggest that the effects on platelets were caused by blockade of elastase activity. Elastase has been reported to modulate coagulation tonus through cleavage of TFPI [10], [12], the physiologic inhibitor of TF [11]. Figure 4D confirms that elastase cleaves TFPI with the appearance of lower mol. wt. bands that are reportedly inactive as anticoagulants [10]; however, cleavage of TFPI was partially or completely blocked when elastase was pre-incubated with Agaphelin at 0.1 µM and 1 µM, respectively. Neutrophils induce coagulation of plasma through release of NETs and activation of the contact pathway [4]–[8]. In order to determine whether Agaphelin affect neutrophil-induced clot formation, inhibitor was incubated with neutrophil, followed by addition of phorbol myristate acetate (PMA) to induced release of procoagulant NETs. The mixture was added to citrated plasma and reactions started with CaCl_2_. Figure 4E shows that addition of neutrophils induces clot in 370 sec while in the presence of PMA clotting time is significantly shortened to 250 sec. However, Agaphelin-treated neutrophil completely loses its pro-coagulant activity. As a control, DNAse also reversed the procoagulant of PMA-activated neutrophils, suggesting that interfering with NETs formation resulted in inhibition of neutrophil-induced coagulation.

### Agaphelin inhibits NETs formation

**Figure 5:**
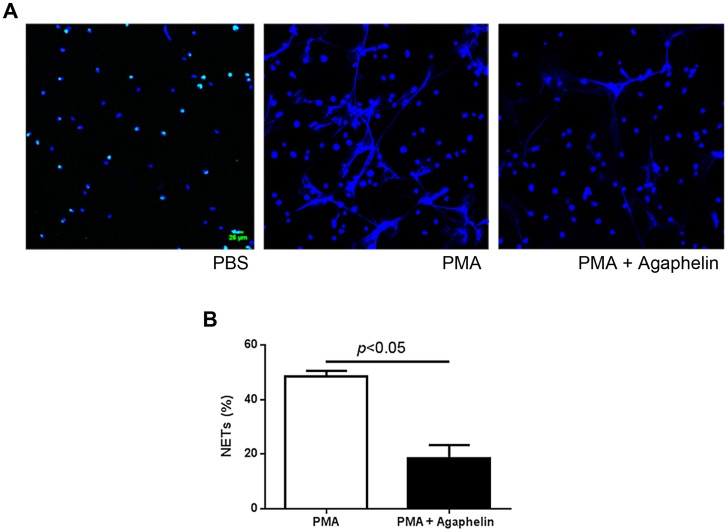
Agaphelin inhibits NETs formation. (A) Adherent neutrophils were incubated with Agaphelin or PBS (control) for 1 hour, and activated with PMA (5 nM, central panel), or PMA plus Agaphelin (right panel) for 3 h at 37°C. Left panel, non-activated neutrophil (no PMA). Formation of NETs was visualized under confocal microscopy, using antibody against citrullinated histone. Representative experiment is shown (4 different donors). B) Quantification. Performed as described in Methods. Sixty five per cent inhibition of NETs formation was attained with 1 µM Agaphelin (*p* = 0.03, *t*-test).

Pharmacologic inhibition of elastase, or mice knockout (KO) for elastase have impaired formation of NETs [38]. To examine the effects of Agaphelin in this response, neutrophils were incubated with Agaphelin for 1 hour. Cells were then exposed to PMA for 3 hrs, and NETs were identified by confocal microscopy using antibodies against citrulinated histone, a marker of NETs formation [4]–[8]. Figure 5, left panel, shows that unstimulated neutrophils do not release NETs. In contrast, 5 nM PMA induces a strong response (central panel), which was dramatically inhibited by Agaphelin (1 µM, right panel). Quantification of results obtained with 4 different donors determined that Agaphelin promoted ∼65% inhibition (p = 0.03, *t*-test, n = 4) of NETs formation (Figure 5B). As a control, addition of DNAse (Dornase alfa, 4 µg/ml) completely abrogated the fluorescence signal associated with NETs (not shown).

### Agaphelin displays anti-inflammatory and antithrombotic activities *in vivo*

**Figure 6:**
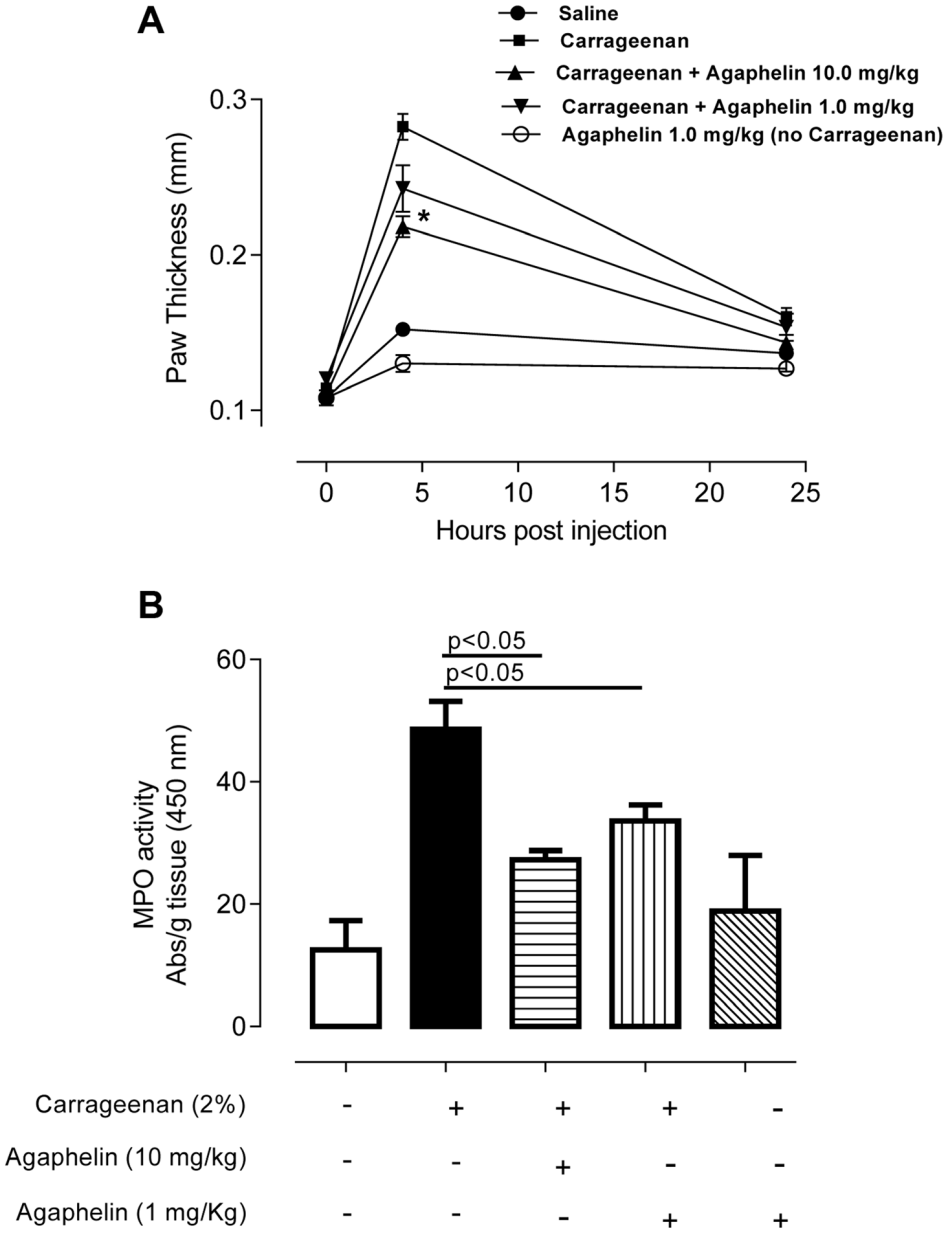
Agaphelin inhibits inflammation and neutrophil accumulation *in vivo*. A) Paw edema in mice. Carrageenan (2%) was administered to mice, in the presence of saline or Agaphelin (1 or 10 mg/Kg). Agaphelin diluted in saline was injected as a control (without carrageenan). Edema formation was evaluated at 0, 4, and 24 h after as increase in paw thickness. B) Neutrophil recruitment in inflamed footpads was evaluated by measuring tissue myeloperoxidase activity, expressed as units of activity/g of tissue, after injection of carrageenan as above. Animals were euthanized 4 hours after. Statistical significance: *, *p*<0.05 or **, *p*<0.01 (one-way ANOVA followed by Tukey's post hoc test; *n* = 5 in each group), compared with carrageenan only.

We evaluated the effects of Agaphelin in a mouse model of acute inflammation induced by carrageenan. When inoculated into the mouse footpad, carrageenan induces an acute inflammatory process characterized by edema formation and intense neutrophil migration. This effect was confirmed here by an increase in paw thickness reaching a maximum 4 h post injection (Fig. 6A). Agaphelin at 1 mg/Kg promoted a non-significant reduction of edema formation. However, in the presence of 10 mg/kg of Agaphelin, paw edema induced by carrageenan was significantly inhibited (*P*<0.05). At 24 h, there were no differences in edema formation in any of the experimental groups. Next, we analyzed carrageenan-induced recruitment of neutrophils in the footpads by measuring tissue MPO activity at 4 h, the time point at which edema reaches a maximum (Fig. 6B). Statistically significant inhibition of MPO activity (30.9%) was observed when 1.0 mg/kg of Agaphelin was co-administered with carrageenan. The inhibition reached 43.2% when 10.0 mg/kg of Agaphelin was co-administered with carrageenan (*P*<0.01). Inoculation of Agaphelin (1 mg/Kg) only without carrageenan (control) did not induce edema or neutrophil accumulation (Fig. 6B).

**Figure 7:**
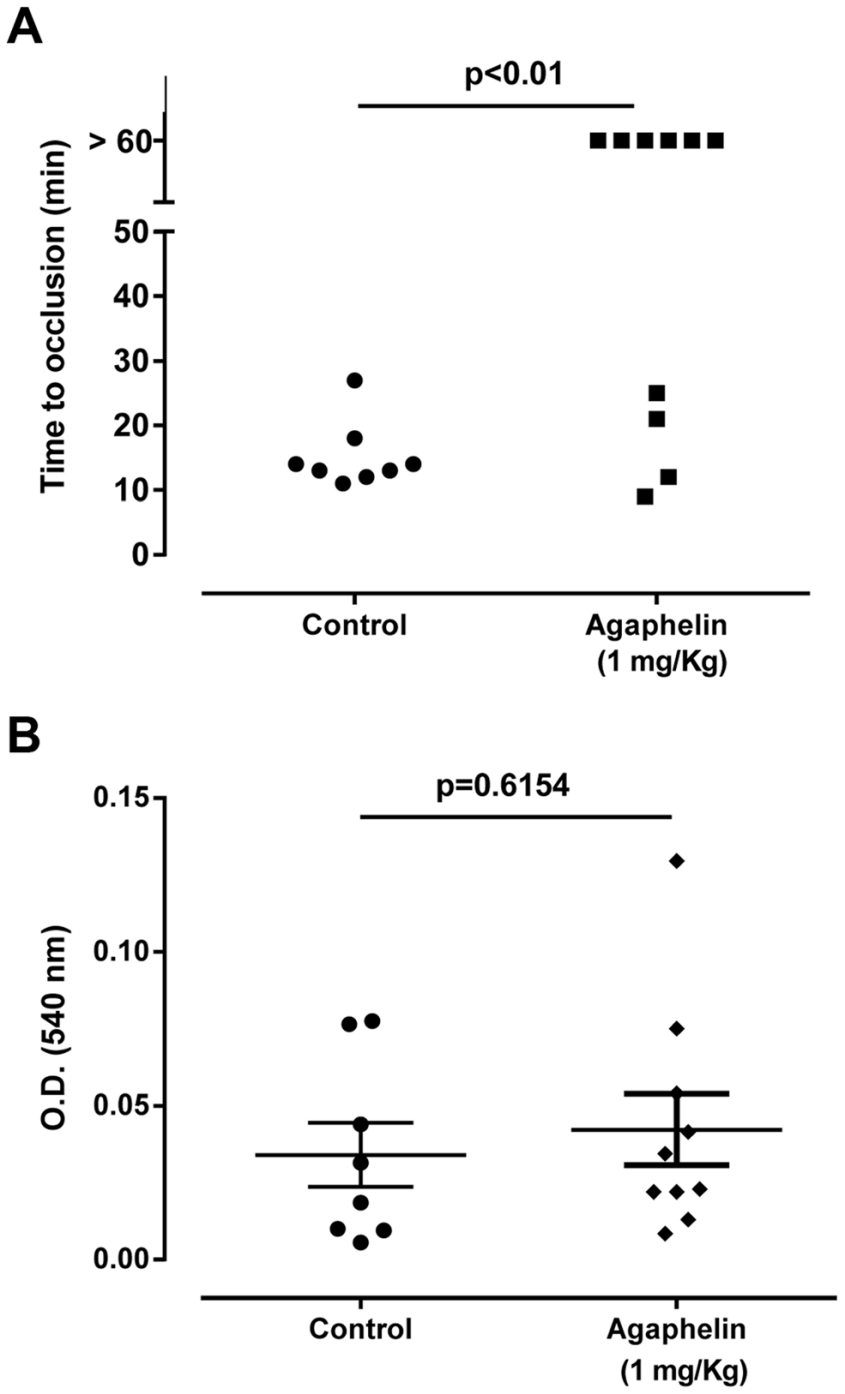
Agaphelin inhibits thrombosis *in vivo*, without impairing hemostasis. A) Arterial thrombosis. A paper filter infused with 7.5% FeCl_3_ was applied to the carotid artery, and blood flow was monitored with a perivascular flow probe for 60 min or until stable occlusion took place. Fifteen minutes before injury, Agaphelin was injected into the caudal veins of the mice. Each symbol represents one animal. *, *p*<0.05 (ANOVA with Dunnett post-test). B) Bleeding time. Bleeding was caused by a tail transection after intravenous injection of Agaphelin at the indicated concentrations. Absorbance at 540 nm (hemoglobin concentration) was used to estimate blood loss. *, *p*<0.05 (ANOVA with Dunnett post-test).

The effect of Agaphelin on thrombus formation *in vivo* was evaluated with FeCl_3_-induced carotid artery thrombosis model in mice. Figure 7A shows that after addition of FeCl_3_, occlusion of the artery occurs in approximately 18 min, as previously reported [39]. In contrast, Agaphelin (1 mg/Kg) promoted a complete inhibition of vessel occlusion after 60 min in 6/10 animals, demonstrating a consistent antithrombotic activity. Agaphelin did not prolong vessels occlusion in 4 animals, which is congruent with variations reported for this technique that involves the lesion size, temperature, concentration of FeCl_3_, and mice strains [40], [41]. To test the effects of Agaphelin in bleeding time, the tail transection method was used. Figure 7B shows that Agaphelin at antithrombotic concentration did not promote bleeding, indicating that it does not impair hemostasis.

## Discussion

To understand how sporozoites affect gene expression in the SG, we employed Affymetrix chips containing approximately 14,900 *A. gambiae* transcripts. Microarray results demonstrate that several SGs genes are upregulated and a few others are downregulated upon infection with *P. falciparum*. Among those upregulated are genes involved in antimicrobial, humoral, and stress responses, chitin and metal metabolism, hemostasis, lipid biosynthesis, transport, signal transduction, regulation of saliva secretion, and chemotaxis, and also other genes described elsewhere [42]–[44]. Interestingly, we discovered that one upregulated gene codes for the protein Agaphelin, a novel monomeric member of the Kazal-family of inhibitors [45]. This family is evolutionarily expanded, since several orthologues were found in other Diptera, including *Aedes* sp and *Culex* sp.

Kazal inhibitors reportedly block several enzymes including subtilisin, granzyme A, elastase, proteinase K, thrombin, trypsin, α-chymotrypsin, and urokinase-type plasminogen activator [34]. Screening assays determined that chemically synthesized Agaphelin inhibits the catalytic activity of neutrophil elastase. This specificity is congruent with Leu in the P1 position of other Kazal inhibitors which exhibit the same inhibitory profile [23], [34], [45]. As a control, another synthetic salivary Kazal-type inhibitor from *Triatoma infestans* (gi 149898841) which does not exhibit Leu in the P1 position did not inhibit elastase or any other enzyme (data not shown). Kinetic experiments revealed that Agaphelin behaves as a slow and tight inhibitor of elastase with a *Ki*∼10 nM. This value is in good agreement with the *K_D_* of 17 nM calculated by SPR experiments. Our results also demonstrated that Agaphelin inhibits neutrophil-associated proteinase-3, which shares 57% homology with elastase. In contrast, it does not affect cathepsin G catalytic activity, which is only 37% homologous. Agaphelin also does not inhibit enzymes involved in coagulation or inflammation, nor does it prolong aPTT and PT. Agaphelin does not promote vasodilation or inhibit bacterial growth, two properties described before for members of the Kazal family of inhibitors [32], [46]. These experiments indicate that Agaphelin, due to its high specificity, is an ideal tool to understand the participation of neutrophil elastase in inflammatory events *in vitro* and *in vivo*.

Elastase exhibits a broad range of substrates and is released rapidly at high concentrations from neutrophil at sites of inflammation. Once released, serine protease may bind to the cell surface and remains relatively inaccessible to inhibitors, in contrast to soluble enzymes [47], [48]. Elastase and other serine proteases reportedly modulate several functions of neutrophils, including chemokine activation and degradation, cell recruitment, receptor activation, activation of lymphocytes, NET formation, and cleavage of adhesion molecules, apoptotic proteins, and anticoagulants [15]. Assays performed with TAXIScan chamber determined that Agaphelin interferes with chemotaxis toward *f*MLP and affected directionality, total path length, and speed of migrating cells. These results suggest that elastase plays a major regulatory role in chemotaxis *in vitro*. This contention is corroborated by previous reports showing that pharmacologic inhibition of elastase by L658758, Eglin C, or SLPI also blocks chemotactic neutrophil functions according to migration induced by PAF, *f*MLP or IL-8 [49]–[51]. While the mechanism is not precisely understood, blockade of cell surface proteinases inhibits intracellular signal for cytoskeletal change and polarization [49].

In agreement with *in vitro* results, Agaphelin reduces paw edema formation triggered by carrageenan and decreases neutrophil infiltration determined by tissue MPO activity, a sensitive marker of neutrophil accumulation. Others have shown that pharmacologic inhibition of elastase with L658758 or Eglin-C is accompanied by inhibition of neutrophil transmigration, based on intravital microscopy of inflamed microvessels [50]. KO mice for elastase also exhibit reduced zymozan-induced leukocyte migration through the cremasteric muscle venules and reduced production of IL-1β, CXCL1, and CCL3 [48]. Moreover, neutrophils from KO mice for both elastase and cathepsin G have impaired recruitment in a subcutaneous air-pouch model of inflammation, or arthritis induced by anti-collagen antibodies [52]. Mechanistically, it has been suggested that elastase inhibitors affect the function of other pro-inflammatory cells, therefore modulating chemokine release by neutrophils and leukocyte infiltration [15]. Elastase also cooperates with cell-surface integrins (α6β1) and PECAM-1, and cleaves E-cadherins, ICAM-1 and VCAM-1, which play an important role in cell-cell contact formation and in mediating neutrophil migration through the perivascular environment [15]. Altogether, inhibition of elastase is potentially associated with blockade of several relevant pathways that may result in diminished neutrophil-mediated inflammation observed here and reviewed elsewhere [15].

Elastase and proteinase-3 potentiate cathepsin G-induced platelet aggregation by a mechanism involving an increase in affinity of integrin α_IIb_ for fibrinogen through limited proteolysis [37]. As expected for an elastase inhibitor, Agaphelin abrogated potentiation of cathepsin G-mediated aggregation by elastase, and this effect may decrease the contribution of platelets to the inflammatory tonus at the site of bite [53]. In addition, elastase cleaves TFPI [10], the physiological inhibitor of the extrinsic pathway [11]. This activity has been suggested to attenuate the anticoagulant effects of TFPI [10], [12]. Our experiments show that TFPI cleavage by elastase is completely abrogated by Agaphelin *in vitro*, and this effect may contribute to shifting the hemostatic balance toward a prothrombotic phenotype *in vivo*.

Neutrophils play a major role in thrombus formation through release of NETs by a complex mechanism named NETosis. While completion of this process may take hours, neutrophils may also rapidly expel NETs (within minutes) [8], [54]. Functionally, NETs contain pro-coagulant TF, promote contact pathway activation, contribute to platelet activation as a scaffold and through histones H3 and H4, and facilitates interaction of elastase with TFPI [4], [5], [7], [8], [12]. Our experiments demonstrate that Agaphelin reduces NETs formation *in vitro*, detected with antibodies against citrulinated histones, indicating that elastase is required for this process. In this context, elastase KO mice or pharmacologic inhibition of intracellular elastase with NEi exhibits impaired NETosis [38], [55]. It has been demonstrated that intracellular release of elastase, degradation of specific histones, and chromatin decondensation are critical for NETs formation by a mechanism synergized by myeloperoxidase and involving reactive oxygen species and the MAP-MEK-ERK pathway [4]–[8], [38]. Of relevance to Agaphelin's mechanism of action—which is a secreted protein—exogenously added elastase inhibitor serpin B1 is internalized, translocates into the nucleus, and inhibits NETs formation [56]. Furthermore, SLPI, another elastase inhibitor, is internalized by monocyte-like cells and found to bind to NF-κB binding sites and to inhibit p65 binding and promote inhibition of TNF-α and IL-8 generation [57].

Agaphelin inhibits arterial thrombus formation induced by FeCl_3_. While Agaphelin prevents vessel occlusion in most animals, 4 of them were not affected by the inhibitor. Conceivably, the mechanism of action of Agaphelin is more subtle than observed with other direct and potent salivary anticoagulants we have tested in this model [39], [58]–[60], besides variations inherent to this technique [40], [41]. Nevertheless, these results are congruent with inhibition of elastase activity, NETs formation, chemotaxis and platelet aggregation observed herein, *in vitro*. In fact, platelets participate in coagulation reactions and thrombus formation [61]–[63]. NETs contribute to thrombosis in baboons, which is prevented by DNase treatment [19]. Moreover, elastase KO mice exhibit less cleavage of TFPI and are resistant to thrombosis *in vivo*. Furthermore, degradation of TFPI by elastase is crucial for controlling the spread of intravascular bacteria [12]. These results suggest that *P. falciparum* infection might also be positively modulated by elastase, and counteracted by Agaphelin. We have also verified that Agaphelin does not promote bleeding, according to the tail transection method. It has become clear that the relative contributions of the intrinsic and extrinsic pathways in thrombosis and hemostasis are different. For instance, targeting components of the contact pathway has emerged as an alternative to prevent thrombosis without increase in bleeding [64]. Because neutrophils play a major role in thrombosis through NETs formation—which is particularly relevant in activation of the contact pathway [18], [64]—our results support the notion that targeting neutrophil functions, and likely other components of the innate immune response, is an alternative strategy to prevent pathologic inflammation and thrombosis [5], [6], [16]–[21]. However, the relative contribution of blockade of neutrophils, platelets, or coagulation by Agaphelin in the inhibition of thrombus formation remains to be determined. With respect to the dose, Agaphelin at 1 mg/kg equals 20 µg per animal (antithrombotic effect) and may reach a maximum theoretical plasma concentration of 1–2 µM which is at least 50 fold above the *K_D_* for elastase (assuming no losses and 1.5-mL volemia). Consistent with our findings, Elafin, a potent elastase inhibitor purified from bronchial secretions, blocks inflammation in several *in vivo* models and is under clinical trial to demonstrate whether it can attenuate myocardial infarction, ischemia-reperfusion injury in patients undergoing coronary bypass surgery [14]. Accordingly, Agaphelin can potentially be used as a drug or as prototype to develop inhibitors of elastase in several pathologic conditions [6], [16]–[21], [29].

On the vector side, Agaphelin is the first salivary antihemostatic to be upregulated by *P. falciparum* infection. In addition, it displays a novel anti-thrombotic mechanism which is mediated by inhibition of neutrophil-associated elastase. Accordingly, Agaphelin may contribute to the repertoire of salivary anti-hemostatics from *An. gambiae*, which includes Anophelin (anticoagulant) [65], apyrase (platelet inhibitor) [2], [3], antigen-5 members (antioxidant) [66], and peroxidase (vasodilator) [67]. SGs are also critical in allowing sporozoites to become infective [68] and might also facilitate *Plasmodium* sp. survival in the skin by blocking the effects of neutrophil-derived elastase and proteinase-3. In fact, human neutrophil elastase is known to degrade the major circumsporozoite protein of the infective stage of *P. vivax* and to interfere with cytoadherance on erythrocytes infected with *P. falciparum* [69]. Therefore, blockage of elastase by Agaphelin could potentially protect the sporozoite during its residency period and transit in the skin. Testing the effect of gene silencing of Agaphelin through siRNA or transgenic approach may potentially determine its role in parasite transmission to the host. Nevertheless, our data suggest that *P. falciparum* induces expression of genes in the invertebrate host that can be used to manipulate the vertebrate host's immune and hemostatic environment, potentially for the parasite's benefit. These results suggest that the parasite changes the local SG milieu to generate a more favorable environment for its development, which could also contribute to increase its infectivity. Upregulation of genes involved in stress response and antimicrobial response also indicates that the presence of the parasite on SGs is not free of costs for the mosquito. Altogether, our results suggest that a notable interplay exist in parasite-vector-host interactions.

## Materials and Methods

### Sporozoite preparation

*An. gambiae* mosquitoes were infected with *P. falciparum* (3D7) by allowing mosquitoes to feed on 14 day-old gametocyte cultures prepared as previously described [70]. Infections in the midgut were assessed by dissecting mosquitoes 9 days post infection and staining midguts with 0.1% mercurochrome solution in water. Infected mosquitoes and uninfected controls were used to isolate midguts at day 9 post infection and SGs at day 20. Dissected tissues were immediately placed on RNAlater solution (Ambion Inc. Austin, TX, USA) and stored at −80°C until further processing. As a technical note, the laboratory infection of *A. gambiae* with *P. falciparum* gametocyte cultures can achieve infection loads of oocysts in the midguts that are higher than those from natural infections that lead to <5 oocysts in the midgut. It is therefore expected that the infection load of the salivary gland with sporozoites would be higher in the case of laboratory infections (9,000–68,000) [71] versus field infections. Nevertheless, there is high variability in the number of sporozoites in salivary glands of field infected Anopheles. For example, it was found in Kenya that salivary glands of naturally infected *A. gambiae* ranged contained from 125 to 79,875 sporozoites. About half of the mosquitos had sporozoite loads higher than 1,000 [72]. This indicates that the laboratory infections of *An. gambiae* with *P. falciparum* can achieve in general high loads of sporozoites in the salivary glands but are still within levels that can be found in nature.

### RNA extraction and T7 RNA amplification

Three pools—each containing whole organs from approximately 20 mosquitos—were used for total RNA extraction using Trizol reagent (Invitrogen, San Diego, CA, USA). For infected organs, we extracted the mosquito and parasite RNA simultaneously. RNA was amplified and labeled using two rounds of linear amplification according to the manufacturer's protocol (GeneChip® two-cycle cDNA synthesis kit; Affymetrix, Santa Clara, CA, USA). RNA quality and purity before and after amplification were assessed by high-resolution electrophoresis using the Agilent 2100 Bioanalyser system (Agilent Technologies, Inc., Santa Clara, CA, USA).

### Labeling and hybridization

Labeled targets from the samples were combined with 2× hybridization buffer, 3 nM B2 control oligo (Affy 900457), and 20× spike (Affy 51214) in DMSO and nuclease-free water, making a final volume of 200 µl for the 48 individual hybridizations to commercial Affymetrix GeneChip *Plasmodium*/*Anopheles* containing both the *P. falciparum* and *An. gambiae* genomes (Cat# 900511; Affymetrix). The hybridization cocktail—including the components listed above—was transferred into the chip and hybridized at a constant temperature of 45°C for approximately 48 h using the approved Affymetrix 640 oven, 500 k.

### Fluidics and scanning

Each chip was filled with 200 µl of wash buffer A, then processed on the fluidics station 450 using a stain mixture of 2× MES stain buffer, 50 mg/ml BSA, 1 mg/ml of streptavidin phycoerythrin, and water to make up 600 µl total volume for each stain and a holding buffer added to make up 600 µl total volume for storage and scanning. Upon completion of the fluidics process, each chip was scanned using the Affymetrix 7Gplus GeneChip scanner to create the image files (dat files).

### Data sharing

All data are MIAME compliant and were deposited on the Gene Expression Omnibus database (GSM465895, GSM465898, GSM465905, GSM465901, GSM465904, GSM465896) [73].

### Quality analysis

GeneChip Operating Software (GCOS v 1.4) was used to convert the image files to cell-intensity data (cel files). All cel files—representing individual samples—were normalized using the scaling method within GCOS and a scaled target of 500 using a *P. falciparum* filter to produce the analyzed files (chp files) along with the report files (RPT) and a pivot table for export into other software. A report was generated from the chp files (along with selected raw data) summarizing various quality and statistical aspects from the chips. From the raw data, the expected signal gradient was produced from the Affymetrix 20× spike in hybridization controls with creX, bioD, bioC, and bioB at 100, 25, 5, and 1.5 pM (supplemental material).

### Data analysis and annotation

The pivot table from all samples, mentioned above, was created including calls, call *P*-value, and signal intensity for each gene. Genes with p values <0.05 and fold change >2 were considered as being significant. The table was imported into GeneSpring GX 7.3 and normalized to a constant value of 500. A hierarchical clustering (condition tree) using a Pearson correlation similarity measure with average linkage was used to produce the dendrogram indicating the cluster from these biological samples. We electronically annotated the entire *P. falciparum* genome using the default settings of Blast2GO [57] and by searching further information on Anoxcel [58] and Biomart [59]. We calculated the correlation between the Affy microarray data and the nanostring data with the Pearson's product moment correlation statistic using the R language [60].

### Nanostring data

Total amplified RNA extracted from midguts and SGs was used to determine mRNA abundance by nanostring technology. In brief, amplified RNA from each sample was allowed to hybridize with the capture and reporter probe and incubated overnight at 65°C according to the nCounter gene expression assay manual (NanoString Technologies, Seattle, WA, USA). After the washes, purified Target/Probe complexes were eluted off and immobilized in the cartridge for data collection carried out in the nCounter digital analyzer. Data were normalized according to the guidelines provided by NanoString Technologies using the nCounter RCC collector worksheet and analyzed as described above.

### Synthesis of Agaphelin

The peptide was synthesized using an automated peptide synthesizer, model 433A (Applied Biosystems, Fullerton, CA, USA) with Fmoc strategy and HBTU/DIPEA as the coupling reagent. Novabiochem Fmoc-Phe-Wang-LL resin (0.20 mmol) (EMD Millipore, division of Merck KGaA, Darmstadt, Germany) was used as the solid phase. The side-chain protecting groups used in synthesis were Trt for Asn, Cys, Gln, and His; OtBu for Glu and Asp; Pbf for Arg; and tBu for Ser, Thr, and Tyr. The coupling reaction time was 1 h, and 4-methylpiperidine (20%)/N-methylpyrrolidone was used to remove the Fmoc group at every step. Peptide resin was washed with N-methylpyrrolidone and dichloromethane and dried *in vacuo* to yield the protected peptide-resin. The peptidyl resin was treated with a cleavage mixture of trifluoroacetic acid/thioanisole/1,2-ethanedithiol/triisopropylsilane (90∶5∶3∶2, v/v/v/v; 40 ml) for 2.5 h to remove protecting groups and peptide from the resin. After filtration of the exhausted resin, the solvent was concentrated *in vacuo* and the residue was triturated with methyl t-butyl ether. The solid peptide was filtered off, washed with methyl t-butyl ether, and vacuum dried. The crude peptide was purified by preparative reversed-phase high-performance liquid chromatography (HPLC), and purity grade was checked by analytical HPLC analyses and mass spectrometry using a matrix-assisted laser desorption ionization time-of-flight mass spectrometer Axima CFR+ (Shimadzu Scientific Instruments. Columbia, MD, USA). Pure fractions were combined, frozen, and lyophilized to afford linear (unfolded) peptide. A second Kazal-type inhibitor from *Triatoma infestans* (gi149898841) was synthesized and refolded as above. Peptide was 94.33% pure with molecular mass 5705.5 da.

### Folding of Agaphelin

Linear, purified peptide Agaphelin (1–58) was dissolved in 6 M guanidin•HCl/200 mM PBS at a concentration of 2 mmol, and the solution was introduced by a Harvard Apparatus “Elite 11” through a syringe, to a 30 times larger volume of the stirred solution of degassed, 50 mM Tris/1 mM EDTA, pH∼8, containing reduced glutathione and oxidized glutathione at concentrations of 1.6 mM and 0.2 mM, respectively. The progress of folding was monitored by HPLC using a gradient of acetonitrile/water with UV monitoring at 215 nm. After 3 h, the reaction mixture was acidified with 2% trifluoroacetic acid to pH 5. The folded peptide was isolated by preparative reverse-phase HPLC and its purity checked by HPLC analyses and mass spectrometry using a matrix-assisted laser desorption ionization time-of-flight mass spectrometer Axima CFR+ (Shimadzu Scientific Instruments). Pure fractions were combined, frozen, and lyophilized to afford pure, folded peptide. Agaphelin molecular mass is 6273 Da (58 amino acids, mature form) with an estimated p*I* 5.09. Extinction coefficient at 280 nm is 3355; A280 nm/cm 0.1% (1 mg/ml), 0.535. Agaphelin was diluted in PBS (1–1.5 mM) and frozen at −80°C.

### Clustal alignment and phylogenetic tree

A sequence similarity search to Agaphelin (gi 118789673) was performed using protein PSI-BLAST against Arthropoda organisms with a threshold of 0.005. Other Kazal domain-containing proteins from *Triatoma infestans* (gi| 149898841, gi| 149898876), *Triatoma brasiliensis* (gi| 116267193), *Tabanus yao* (gi| 161897822, gi| 241914365, gi| 241914367), *Hybomitra bimaculata* (gi| 94730670, vasotab), *Dipetalogaster maxima* (gi| 4033530, dipetalogastin), *Rhodnius prolixus* (gi| 730939, rhodniin), *Hirudo medicinalis* (gi| 124043, bdellin B-3), and *Hirudo nipponia* (gi| 14279682, bdellin-KL) were also added. Sequences from the nonreduntant (NR) protein database of the National Center for Biotechnology Information (NCBI) are represented by six letters followed by the NCBI gi| accession number. The six letters derive from the first three letters of the genus and the first three letters from the species name. Protein sequences were aligned by the ClustalX software program [74]. The phylogenetic tree was performed with the Mega package [75] after 10,000 bootstraps with the neighbor joining algorithm using the poisson model and pairwise deletion.

### Structural modeling and alignment

The sequence of the Agaphelin protein (AGAP007907-PA) was downloaded from Vectorbase [76]. The presence and location of a signal peptide was predicted using a SignalP 4.1 Server [77]. The signal peptide was removed from the protein sequence, and the structure was homology modeled using Swiss-Model Automated Mode [78], Phyre2 [79], and I-Tasser [80], [81]. The structures were then aligned to its best template (*T. infestans* Infestin 4, PDBID: 2ERW) and to each other using the multiple alignment of protein structures and consensus identification server [82]. Secondary structure prediction was done for each individual model using DSSP, and the percent composition of each secondary structure motif was calculated for the three models [83]. The aligned structures of Agaphelin and Infestin 4 were further processed using the PyMOL molecular graphics system (v. 1.4.1; Schrödinger, LLC, San Diego, CA, USA).

### Analytical SEC-MALS HPLC

The solution state of the peptide was analyzed using analytical size-exclusion chromatography with on-line multi-angle light scattering (SEC-MALS). Chromatography was performed on an Alliance HPLC system (Waters Corporation, Milford, MA, USA) connected in series to a multi-angle Dawn Heleos light-scattering detector and a quasi-electric light scatter detector (Wyatt Technology, Santa Barbara CA, USA).

Protein separation was done on a TSKgel G2000SW×l column (Tosoh Bioscience, King of Prussia, PA, USA). The column was equilibrated in mobile phase consisting of 1.04 mM KH_2_PO_4_, 2.97 mM Na_2_P0_4_•7 H_2_0, 308 mM NaCl, 0.02% azide, pH 7.4, and the samples were run using an isocratic elution at 0.5 ml/min. A gel filtration standard (Bio-Rad, Hercules, CA, USA) was run for size comparison as well as a 125-µg injection of BSA for configuration of the MALS data. The HPLC data was analyzed with Empower software and the light scattering data with Astra software.

### Circular dichroism

The circular dichroism spectrum (CDS) of the peptide was recorded on a Jasco J-815 CD spectropolarimeter over the wavelength range of 260–190 nm. Data were collected using a slit bandwidth of 1.0 nm and a signal averaging time of 1.0 second in a 1-mm quartz cuvette. Raw data measured in millidegrees were converted into ellipticity (degrees cm^2^ dmol^−2^). CDS was deconvoluted using the DichroWeb interface (http://dichroweb.cryst.bbk.ac.uk) [84]. Results having a normalized root mean square of ≤0.3 were used to calculate secondary structure composition.

### Serine protease inhibition screen

The screen was performed as previously described [58], [85]. Except for the enzyme concentrations, all the rest was kept constant. The following assay concentrations were used. Thrombin (0.01 nM), α-chymotrypsin (0.03 nM), plasmin (0.8 nM), and chymase (0.45 nM) were purchased from Sigma-Aldrich (St. Louis, MO, USA).

Human skin β-tryptase (0.01 nM) was purchased from Promega (Madison, WI, USA), FXa (0.33 nM) from EMD Biosciences, Inc. (San Diego, CA, USA), FXIIa (0.1 nM) from Haematologic Technologies Inc. (Essex Junction, VT, USA), kallikrein (0.04 nM) from Fitzgerald Industries International (Concord, MA, USA), elastase (0.06 nM) from Elastin Products Company, Inc. (Owensville, MO, USA), and Cathepsin G (5.3 nM), FXIa (0.06 nM), urokinase plasminogen activator (uPA; 0.25 nM), and tissue plasminogen activator (t-PA; 0.02 nM) were purchased from Molecular Innovations (Southfield, MI, USA). Matriptase (0.03 nM) was obtained from R&D Systems (Minneapolis, MN, USA), proteinase 3 (11 nM) from Merck-Millipore (Billerica, MA, USA), and sequencing-grade trypsin (0.1 nM) was purchased from Roche Molecular Biochemicals (Indianapolis, IN, USA).

### aPTT and PT

aPTT and PT were evaluated on a STart 4 stago coagulometer (Diagnostica Stago, Parsippany, NJ, USA). Freeze-dried, citrated, normal human plasma was resuspended in ultra-pure water. For the aPTT, plasma (50 µl) was incubated with 7 µl Agaphelin or PBS (control) in appropriate cuvettes and placed in the coagulometer for 2 min at 37°C. Then, 50 µl of pre-warmed aPTT reagent (STA PTT; Diagnostica Stago, Asnières sur Seine, France) was added and incubated for an additional 2 min. CaCl_2_ (50 µl at 25 mM) was added to start reactions. For PT, plasma (50 µl) was incubated with Agaphelin or PBS (control) and placed in the coagulometer for 2 min at 37°C. Then, 100 µl of the PT reagent (NEOplastine CI plus; Diagnostica Stago) was added. Time for clot formation was recorded in duplicate.

### Surface plasmon resonance (SPR)

SPR instrumentation (XPR36), GLC sensor chip, and amine coupling reagents containing N-hydroxysulfosuccinimide (sulfo-NHS), N-(3-dimethylaminopropyl)-N-ethylcarbodiimide hydrochloride (EDAC), and ethanolamine HCl were obtained from Bio-Rad. Synthetic refolded Agaphelin was diluted to a concentration of 100 µg/ml in NaOAc buffer (10 mM, pH 4.5) and coupled to the surface of a GLC chip using the manufacture's amine-coupling chemistry as described in the XPR36 system manual. Briefly, the surface of the sensor chip was first activated with EDC/NHS, followed by addition of the peptide. The surface was blocked using ethanolamine. Employing these conditions, surfaces containing densities of 408.27 resonance units of peptide were generated. Surface regeneration was done using 10 mM HCl. Sensograms were recorded and normalized to a baseline of 0 resonance units. Equivalent volumes of elastase were also injected over a mock, no-protein, blocked surface to serve as a blank sensogram for subtraction of bulk refractive index background. Sensograms were analyzed for fitting using XPR36 software (Bio-Rad). A Langmuir single-site binding model (A+B = AB) was used for analysis of interaction of the Kazal peptide and elastase surface.

### Chemotaxis assay

HL-60 cells (human promyelocytic cells) (American Type Culture Collection, Manassas, VA, USA) were maintained in an undifferentiated state in RPMI 1640 media containing 10% fetal bovine serum and 25 mM Hepes at 37°C in a humidified 5% CO_2_ atmosphere. HL-60 cells differentiated in culture medium containing 1.3% DMSO for 5 days before experiments. The EZ-TAXIScan chamber (Effector Cell Institute, Tokyo, Japan) was assembled as described by the manufacturer. HL-60 cells were incubated for 1 h with Agaphelin at 37°C. Cell migration was recorded every 15 s for 30 min at 37°C in a humidified environmental chamber. Coverslips and chips used in the chamber were coated with 1% BSA at room temperature (RT) for 1 h. All glass coverslips were ultrasonicated and washed before use. Cell migration analysis was conducted with DIAS software.

### Platelet aggregation

Platelet-rich plasma was obtained by plateletpheresis from medication-free platelet donors at the Department of Transfusion Medicine (NIH blood bank). Washing of platelets and platelet aggregation were performed in a Lumi-aggregometer (Chronol-Log, Haverstown, MD, USA) as described [86], [87].

### Vasodilation

Contraction of rat aortic ring preparations by U 46619 was measured isometrically and recorded with transducers from Harvard Apparatus Inc. (Holliston, MA, USA) as reported [88]. A modified Tyrode solution was prepared with the addition of 5 mM Hepes; pH was adjusted to 7.4, and the solution was oxygenated by continuous bubbling of air throughout the assays. In the first assay, aortic rings were suspended in a 0.5-ml bath kept at 36°C; they were pre-constricted by 100 nM U-46619 before addition of Agaphelin to give final concentrations of 1 µM, or SG homogenates of *R. prolixus* (0.04 of one pair of glands/ml, with an approximate final concentration of nitrophorins of 1 µM as positive control) [89]. Additions to the bath were never greater than 5% of the volume of the bath.

### TFPI cleavage

In a PCR tube, Agaphelin at different concentrations (0.1 µM, and 1 µM) was incubated with 1 µg of TFPI (R&D Systems), in a final volume of 20 µl in PBS, followed by addition of 1 µl PBS (control) or human neutrophil elastase (Molecular Innovations; final concentration: 0.8 µg/ml). After 2 h at RT, reactions were stopped by addition of Laemmli buffer (supplemented with dithiothreitol) and boiled for 5 min. Proteins were separated by 4% to 12% NuPAGE (MES buffer); gels were stained with Coomassie Blue R-250 and destained with 40% methanol. See Blue molecular weight (mol. wt.) markers were used (Invitrogen).

### Neutrophils isolation

Whole blood (5 mL collected in 0.5 mL of 3.2% sodium citrate) from healthy donors was diluted in an equal volume of PBS, layered over 5 mL of HISTOPAQUE solution (10771, Sigma Aldrich), and centrifuged for 40 min, at 400×g, at room temperature. The lower interphase having granulocytes was collected and transferred to a 15-ml Falcon tube and resuspended in 10 ml ammonium chloride lysis buffer (1.7 M NH_4_Cl, 0.1 M KCO_3_, 1 mM EDTA) to lyse red blood cells. Lysis was carried out twice followed by centrifugation for 10 min at 400×g. Neutrophils were washed with PBS and resuspended at 1×10^6^ cells/mL in high glucose DMEM (GIBCO). Neutrophils were kept on ice.

### Neutrophil-induced coagulation

Purified neutrophils (500 µl, 1×10^5^/ml, in RPMI without serum) were incubated with RPMI, Agaphelin (1 µM) or DNAse (Dornase alfa, Genentech Inc. 4 µg/ml) at 37°C for 1 hr, prior stimulation with RPMI (negative control), or 50 nM PMA (at 37°C for 3 h). Then 50 µl of this suspension was added to 50 µl of fresh plasma (collected in citrate) in a coagulometer cuvette (KC4 Delta Coagulometer, Tcoag Ireland Limited, Wicklow, Ireland). Reactions were started by addition of 12.5 mM CaCl_2_ (final concentrations).

### NETs formation

5×10^4^ neutrophils (in 100 µl DMEM) treated with PBS or with Agaphelin (1 µM) were seeded onto 13 mm cover-slips (Glasscyto) and incubated for 1 h, at 37°C, prior to stimulation with Phorbol 12-myristate 13-acetate (PMA), 5 nM (Sigma Chemical Co) for 3 h in DMEM. Cells were fixed with 500 µl of 4% paraformaldehyde (final concentrations) for 10 min, washed 3 times with PBS and incubated for 10 min with blocking solution (PBS, 10%FBS, 5 mg/ml BSA). Samples were incubated with goat polyclonal anti-human histone H3 antibody at 1∶50 (Abcam, 5103) diluted in blocking solution. Samples were washed 3 times with blocking solution followed by 2 h incubation with rabbit anti-goat IgG labeled with Alexa 488 at 1∶500 (Molecular Probes) diluted in blocking buffer and Hoechst 33342 at 1∶1000 diluted in blocking buffer. NETs were visualized under a confocal microscope (Leica, Confocal Microscope LEICA DMI4000 TCS SPE, 20×). Images analyses were performed using Image J software (NIH). NETs were quantified according to Farley et al [56]. Briefly, NETs were identified on digitalized images as Hoechst-positive fibrils emanating from cells with an overall length at least twice as long as the cell diameter and were counted for at least two fields of view per variable. Results were expressed as the percentage of NETs/total number of granulocytes.

### Paw edema

Female C57BL/6 mice weighing 20–30 g (6–8 weeks old) were used. Experimental protocol reference number 276 was approved by the IACUC/Ethical Committee of the Universidade Federal do Triângulo Mineiro. The carrageenan-induced hind paw inflammation model was used to investigate the anti-inflammatory role of Agaphelin. Prior to each injection, the basal footpad thickness of each mouse was recorded using a caliper (Mitutoyo America Corp., Aurora, IL, USA). Subsequently, 40 µl of carrageenan (2% in saline) was administered by intraplantar injection in each posterior footpad in the absence or presence of two different concentrations of LPS-free Agaphelin. As control, groups of mice received the same volume of saline (vehicle) in the presence of Agaphelin only. As an index of edema formation, paw thickness was measured at 4 and 24 h post injection. Each data point is the mean±SD of four paws.

### Tissue myeloperoxidase (MPO) assay

To measure the presence of neutrophils in the paw, activity of MPO in paw tissues was measured as previously described [90]. Briefly, paw tissues from the different groups were homogenized with an electrical tissue homogenizer (30,000 rpm) in 0.5 mL ice-cold buffer (0.1 M NaCl, 0.08 M NaPO_4_, 0.015 M EDTA), pH 4.7, and centrifuged at 3000 rpm for 15 min at 4°C. The pellet was subjected to hypotonic lysis with 200 µl of 0.2% NaCl solution (0.1 M) for 30 s followed by addition of an equal volume of a solution containing 0.2% NaCl). After centrifugation at 3000 rpm for 15 min, the pellet was resuspended in 0.5 mL of 0.05 M NaPO_4_ buffer, pH 5.4, containing 0.5% hexadecyltrimethylammonium bromide (Sigma-Aldrich, EUA) and re-homogenized. The homogenate was frozen and thawed three times and then centrifuged again at 12 000 rpm for 15 min at 4°C. MPO activity in the re-suspended pellet was assayed by measuring in a spectrophotometer the change in absorbance at 450 nm using 25 µL of tetramethylbenzidine 1.6 mM (BD pharmigen) and hydrogen peroxide (0.5 mM). A unit of MPO activity was defined as that converting 1 µmol of hydrogen peroxide to water in 1 min at 22°C.

### FeCl_3_-induced artery thrombosis

BALB/c mice were anesthetized with intramuscular xylazine (16 mg/kg) followed by ketamine (100 mg/kg). Experimental protocol number IBQM081-05/16 was approved by IACUC/Ethical Committee of the Universidade Federal do Rio de Janeiro. The right common carotid artery was isolated through a midline cervical incision, and blood flow was continuously monitored using a 0.5-VB Doppler flow probe coupled to a TS420 flow meter (Transonic Systems, Ithaca, NY, USA) as described [58]. Fifteen min before induction of thrombosis, animals were injected in the tail vein with 50 µl Agaphelin (1 mg/kg) or vehicle (PBS). Thrombus formation was induced by applying a piece of filter paper (1×2 mm) saturated with 7.5% FeCl_3_ solution on the adventitial surface of the artery for 3 min. After exposure, the filter paper was removed and the vessel was washed with sterile normal saline. Carotid blood flow was continuously monitored for 60 min or until complete occlusion (0 flow for at least 10 s) occurred. ANOVA using Tukey as a multiple comparison post-test was used. *P*≤0.05 was considered statistically significant.

### Tail bleeding assay

Mice were anesthetized with intramuscular xylazin (16 mg/kg) followed by ketamine (100 mg/kg) and injected intravenously with PBS or Agaphelin (1.0 mg/kg) in a 100 µl volume. After 15 min, the distal 2-mm segment of the tail was removed and immediately immersed in 40 ml distilled water warmed to 37°C. Samples were properly homogenized and absorbance determined at 540 nm to estimate hemoglobin content. No animal was allowed to bleed for more than 30 min.

### Bacterial strains

Bacterial strains were obtained either from American Type Culture Collection or Eurofins (Chantilly, VA, USA). They were maintained at −80°C in nutrient broth, brain-heart infusion broth, or tryptic soy broth, all with a final concentration of 20% glycerol (v/v). For the experiment, strains were thawed and streaked onto agar media plates for single colonies (1.5% w/v agar); colonies were then picked for liquid culture. Liquid cultures were grown on cation adjusted MHB. Minimum inhibitory concentration inocula were prepared by diluting cultures to achieve a cell density of 1×10^6^ cells/ml.

### Minimum inhibitory concentration (MIC)

Broth microdilution MIC testing was performed by Emeryville Pharmaceutical Services (Emeryville, CA, USA) using the procedure described by NCCLS publication M7-A6. The highest concentration of Agaphelin peptide tested was 512 µg/ml, and the recorded MIC values were the lowest concentration of the test article that completely inhibited growth, which was checked visually after 24 h of incubation. Each strain was tested against ciprofloxacin (1024-0 ng/ml) for quality control.

### Minimum bactericidal concentration (MBC)

Agar dilution plate count MBC was performed by Emeryville Pharmaceutical Services. MBC values were determined using the procedure described in NCCLS publication M26-A. The highest concentration of peptide tested was 512 µg/ml, and the MBC values recorded were the lowest concentration of each test article in which the plate count was at least 3 standard deviations below that representing 0.1% of the final inoculum count.

### Ethics statement

All treatments were performed and conducted in accordance with the NIH guidelines for the welfare of experimental animals and approved by the Ethical Committee of the Federal University of Triângulo Mineiro (Number#276) and Federal University of Rio de Janeiro (IBQM081-05/16).

### Statistical analysis

Results are expressed as means ± SE. Statistical differences among the groups were analyzed by *t*-test or ANOVA using a multiple comparison post-test. Significance was set at *p*≤0.05 (GraphPad Prism Software, San Diego, CA, USA).
